# The role of ArlRS in regulating oxacillin susceptibility in methicillin-resistant *Staphylococcus aureus* indicates it is a potential target for antimicrobial resistance breakers

**DOI:** 10.1080/22221751.2019.1595984

**Published:** 2019-03-29

**Authors:** Jinna Bai, Xiaoyi Zhu, Keqing Zhao, Yingjie Yan, Tao Xu, Jiaxue Wang, Jinxing Zheng, Wei Huang, Le Shi, Yongpeng Shang, Zhihui Lv, Xiaofei Wang, Yang Wu, Di Qu

**Affiliations:** a Key Laboratory of Medical Molecular Virology of Ministries of Education and Health, Department of Medical Microbiology and Parasitology, School of Basic Medical Sciences, Shanghai Medical College of Fudan University, Shanghai, People’s Republic of China; b Department of Otorhinolaryngology-Head and Neck Surgery, Eye & ENT Hospital, Shanghai Key Clinical Disciplines of otorhinolaryngology, Fudan University, Shanghai, People’s Republic of China; c Key Laboratory of Medical Molecular Virology, Huashan Hospital, Shanghai Medical College of Fudan University, Shanghai, People’s Republic of China; d Department of Laboratory Medicine, Hangzhou Medical College, Hangzhou, Zhejiang, People’s Republic of China; e Department of Infectious Diseases and the Key Laboratory of Endogenous Infection, Shenzhen Nanshan People's Hospital of Shenzhen University, Shenzhen, People’s Republic of China; f Medical Clinic, Hangzhou Haiqin Sanatorium, Hangzhou, Zhejiang, People’s Republic of China

**Keywords:** *Staphylococcus aureus*, two-component signal transduction system, ArlRS, *spx*, oxacillin

## Abstract

Methicillin-resistant *Staphylococcus aureus* (MRSA), also known as oxacillin-resistant *S. aureus,* is a leading cause of community and hospital associated infections globally. In this work, we found that deletion of the *arlRS* two-component system genes in the USA300 and USA500 strains resulted in increased susceptibilities to oxacillin (8–16-fold decrease in minimal inhibitory concentrations). In USA300Δ*arlRS,* transcriptional levels of *mecA* or *blaZ* showed no obvious change, while mRNA levels of *spx* showed a 4-fold decrease at 4 h and a 6.3-fold decrease at 10 h. Overexpression of *spx* in Δ*arlRS* restored oxacillin resistance to a similar level in USA300. In addition, gel shift assay showed that the recombinant ArlR bound to *spx* promoter region. Furthermore, silencing of *spx* led to a significant increase of oxacillin susceptibility in multiple MRSA isolates. Our results indicate that ArlRS plays a strong role in regulating oxacillin resistance in MRSA strains, which involves direct modulation of *spx* expression. Moreover, oritavancin showed inhibition to ATPase activity of the recombinant histidine kinase ArlS (IC_50_ = 5.47 μM). Oritavancin had synergy effect on oxacillin activity against the MRSA strains in both planktonic and biofilm state. Our data suggest that ArlRS is an attractive target for breaking antimicrobial resistance of MRSA.

## Introduction

*Staphylococcus aureus* is a major pathogen causing both community-acquired and hospital-acquired infections globally [1]. The emergence and prevalence of community-associated methicillin-resistant *S. aureus* (CA-MRSA) and healthcare-associated methicillin-resistant *S. aureus* (HA-MRSA), e.g. the predominant clones USA300 [2] and USA500 [3], have attracted attentions because of their virulence and antibiotic resistance, especially their high-level resistance to the β-lactam antibiotics [4].

MRSA, also known as oxacillin-resistant *S. aureus*, is defined as an oxacillin minimum inhibitory concentration (MIC) of greater than or equal to 4 mg/L [5]. Oxacillin is a β-lactam antibiotic targeting penicillin-binding protein 2 (PBP2). Oxacillin resistance involves multiple factors. Most MRSA strains possess a *mecA* gene that encodes an alternative form of PBP2 called PBP2a (or PBP2′). PBP2a can take over the transpeptidation function of PBP2, but it has a lower penicillin-binding affinity and is resistant to the action of oxacillin [6]. PBP2a synthesis is modulated by the transcriptional regulator MecI and the signal transduction protein MecR1, which are encoded by *mecI* and *mecR1* genes located adjacent to *mecA* on the staphylococcal chromosome [7,8]. MecI and MecR1 share high protein sequence similarity with BlaI and BlaR1 [9], respectively, which may also have the function of regulating PBP2a expression. Thus, in many clinical isolates of MRSA, a plasmid carrying *blaI* and *blaR1* genes can encode proteins modulating PBP2a expression [10,11]. Beside the main mechanism above, oxacillin resistance in *S. aureus* clinical isolates has been reported to involve other factors, including FemAB (peptidoglycan synthesis), Llm (autolytic activity related protein), Sar, Agr, SigB [12–15] (global regulators), etc.

The two-component signal transduction system ArlRS is a global regulator of *S. aureus* virulence, modulating the extracellular proteolytic activity, bacterial autolysis, capsule formation and production of virulence factors [16–21]. Recent studies has found that ArlRS regulates *S. aureus* cell aggregation [22] and is vital for *in vivo* catheter associated biofilm formation by *S. aureus* [23]. However, whether ArlRS is involved in the regulation of oxacillin resistance remains unclear.

Here, we demonstrate for the first time that ArlRS plays an important role in the regulation of oxacillin resistance in MRSA strains, mainly through the direct modulation of *spx* expression. Besides, oritavancin can inhibit ArlS kinase activity and it has synergetic effect on oxacillin activity against MRSA strains.

## Materials and methods

### Bacterial strains, plasmids, growth media and antibiotics

Bacterial strains and plasmids used in this study are listed in Table 1. The MRSA strains were collected from Zhongshan Hospital of Fudan University and identified by the VITEK 2 system (bioMerieux SA, Lyon, France).

**Table 1.** Bacterial strains and plasmids used in this study.

Bacterial Strains / Plasmid	Description	Source or Reference(s)
Bacterial Strains		
USA300 FPR3757	a MRSA strain (GenBank Accession Number: NC_007793)	[24]
USA300-*arlS*	USA300 FPR3757 with a transposon insertion in *arlS* gene	This study
USA300-*arlR*	USA300 FPR3757 with a transposon insertion in *arlR* gene	This study
USA300 TCH1516	a MRSA strain (GenBank Accession Number: NC_010079)	[2]
USA300 pMX6	USA300 transformed with the plasmid pMX6	This study
USA300-pMX-*spx*	USA300 transformed with the plasmid pMX6-*spx*	This study
USA300-P*mgrA*	USA300 transformed with plasmid pCM29-*mgrA*	This study
USA300-P*spx*	USA300 transformed with plasmid pCM29-*spx*	This study
USA300Δ*mgrA*	an *mgrA* knockout mutant of USA300 TCH1516	This study
USA300Δ*arlRS*	an *arlRS* knockout mutant of USA300 TCH1516	This study
PCN	USA300Δ*arlRS* transformed with plasmid pCN51	This study
PCN*arlRS*	USA300Δ*arlRS* complemented with plasmid pCN51-*arlRS*	This study
PCN-*mgrA*	USA300Δ*arlRS* complemented with plasmid pCN51-*mgrA*	This study
PCN-*spx*	USA300Δ*arlRS* complemented with plasmid pCN51-*spx*	This study
PCN-*trfA*	USA300Δ*arlRS* complemented with plasmid pCN51-*trfA*	This study
Δ*arlRS*-P*mgrA*	USA300Δ*arlRS* transformed with plasmid pCM29-*mgrA*	This study
Δ*arlRS*-P*spx*	USA300Δ*arlRS* transformed with plasmid pCM29-*spx*	This study
USA500 2395	a MRSA strain (GenBank Accession Number: CP007499)	[3]
USA500Δ*arlRS*	an *arlRS* knockout mutant of USA500 2395	This study
USA500-pMX-*spx*	USA500 transformed with the plasmid pMX6-*spx*	This study
PRB	USA500Δ*arlRS* strain introduced with plasmid pRB475	This study
PRB-*arlRS*	USA500Δ*arlRS* strain complemented with plasmid pRB475-*arlRS*	This study
234	a human clinical MRSA isolate	This study
234-pMX	234 strain introduced with plasmid pMX6	This study
234-pMX-*arlR*	234 strain complemented with plasmid pMX6-*arlR*	This study
234-pMX-*arlS*	234 strain complemented with plasmid pMX6-*arlS*	This study
234-pMX-*spx*	234 strain transformed with the plasmid pMX6-*spx*	This study
15098	a human clinical MRSA isolate	This study
15098-pMX	15098 strain introduced with plasmid pMX6	This study
15098-pMX-*arlR*	15098 strain complemented with plasmid pMX6-*arlR*	This study
15098-pMX-*arlS*	15098 strain complemented with plasmid pMX6-*arlS*	This study
15098-pMX-*spx*	15098 strain transformed with the plasmid pMX6-*spx*	This study
184749	a human clinical MRSA isolate	This study
184749-pMX	184749 strain transformed with the plasmid pMX6	This study
184749-pMX-*arlR*	184749 strain transformed with the plasmid pMX6-*arlR*	This study
184749-pMX-*arlS*	184749 strain transformed with the plasmid pMX6-*arlS*	This study
184749-pMX-*spx*	184749 strain transformed with the plasmid pMX6-*spx*	This study
**Plasmid**		
pCN51	a shuttle vector; Amp^r^ in *E. coli* and Em^r^ in *S. aureus*	[25]
pCN51-*arlRS*	the *arlRS* genes and the promoter region in USA300 TCH1516 strain cloned into pCN51	This study
pCN51-*mgrA*	the *mgrA* gene cloned downstream of the cadmium chloride (CdCl_2_) inducible promoter of pCN51	This study
pCN51-*spx*	the *spx* gene cloned downstream of the CdCl_2_-inducible promoter of pCN51	This study
pCN51-*trfA*	the *trfA* gene cloned downstream of the CdCl_2_-inducible promoter of pCN51	This study
pMX6	an ATc-inducible asRNA-expressing plasmid; Amp^r^ in *E. coli* and Cm^r^ in *S. aureus*	[26]
pMX6-*arlR*	pMX6 expressing asRNA of *S. aureus arlR*	This study
pMX6-*arlS*	pMX6 expressing asRNA of *S. aureus arlS*	This study
pMX6-*spx*	pMX6 expressing asRNA of *S. aureus spx*	This study
pKOR1	a temperature-sensitive shuttle plasmid for gene knockout in *S. aureus*; Amp^r^ in *E. coli* and Cm^r^ in *S. aureus*	[27]
pKOR1-*arlRS*	the plasmid for in-frame deletion of *S. aureus arlRS* genes	This study
pRB475	a derivative of the shuttle vector pRB473, Km^r^ in *E. coli* and Cm^r^ in *S. aureus*	This study
pRB475-*arlRS*	the *arlRS* gene and the promoter region in USA300 TCH1516 strain cloned into pRB475	This study
pCM29	a GFP expression shuttle vector; Amp^r^ in *E. coli* and Cm^r^ in *S. aureus*	[28]
pCM29-*spx*	GFP promoter replaced with P*spx* in pCM29	This study
pCM29-*mgrA*	GFP promoter replaced with P*mgrA* in pCM29	This study
pET28a(+)	An isopropyl-1-thio-b-D-galactopyranoside -inducible protein-expressing plasmid; Km^r^	[29]
pET*-arlR*	the *arlR* gene cloned into pET28a(+)	This study

Tryptic soy broth (TSB, Oxoid, Cambridge, UK) were used for *S. aureus* cultivation. Mueller-Hinton Broth and Mueller-Hinton agar were used for antibiotic susceptibility tests. Media were supplemented with erythromycin (10 µg/ml), ampicillin (100 µg/ml) or chloramphenicol (10 µg/ml), when appropriate for purposes of selection. Oritavancin diphosphate (dissolved in DMSO at 10 mM) was purchased from MedChemExpress China. Oxacillin was purchased from Sangon BiotechCo., Ltd (Shanghai, China).

### Construction of gene knockout mutants and complementation strains

The *arlRS* genes in methicillin-resistant *S. aureus* USA300 TCH1516 strain (locus tag: USA300HOU_1349 and USA300HOU_1350) and USA500 2395 strain (locus tag: CH51_07425 and CH51_07430) were deleted using the temperature-sensitive vector pKOR-1 [27]. Then the regions flanking *arlRS* gene were amplified by PCR and inserted into pKOR-1. Primers for PCR were designed according to the genomic sequence of *S. aureus* TCH1516 strain, and the sequences are listed in **Supplementary Table 1**. The recombinant plasmid, designated pKOR-*arlRS*, was transformed to *Escherichia coli* DC10B strain then into *S. aureus* USA300 and USA500 strains by electroporation respectively. A procedure for allelic displacement of the *arlRS* genes was performed as previous described [30,31]. The mutants, designated USA300Δ*arlRS* and USA500Δ*arlRS*, were verified by PCR, RT–PCR and direct sequencing. The vector pCN51 [25] and pRB475 were used for *arlRS* complementation. The DNA fragment of the *arlRS* genes with their promoter region were amplified by PCR and inserted into the vectors. The resulting plasmids were transformed by electroporation into USA300Δ*arlRS* and USA500Δ*arlRS*, forming complementary strains PCN*arlRS* and PRB*arlRS*, respectively.

### Construction of the gene silencing strains and the gene overexpressing strains

To silence the *arlS*, *arlR* or *spx* (locus tag: USA300HOU_0955) gene in *S. aureus* strains, the shuttle plasmid pMX6 [26] with the paired termini 7 segment (which can form a hairpin structure) was used for constructing asRNA expression vectors. The expression plasmid of asRNA*_arlR_* or asRNA*_spx_* (named pMX6-*arlR* or pMX6-*spx*) was constructed by first amplifying the predicted Shine-Dalgarno (SD) sequence plus ∼100 nt downstream of the start codon of each gene and then inserting the fragment in the reverse direction between EagI and BglII sites downstream of the anhydrotetracycline (ATc) inducible promoter in pMX6. As for generating the expression plasmid of *arlS* asRNA (named pMX6-*arlS*), a ∼120 nt sequence downstream from the start codon of the *arlS* gene was amplified.

To overexpress *spx* in *S. aureus* strains, the coding sequence of *S. aureus spx* gene were amplified with the primers SA-spx-RBS-F and SA-spx-RBS-R (**Supplementary Table 1**), and was inserted between the BamHI and EcoRI sites downstream of the cadmium chloride (CdCl_2_) inducible promoter of the shuttle plasmid pCN51, generating pCN51-*spx*.

The sequences of all the asRNA expression plasmids and gene overexpression plasmids were verified by DNA sequencing. Then each plasmid was transformed into *S. aureus* strains by electroporation.

### Detection of bacterial growth curves

The growth curves of the *S. aureus* strains were determined by measuring the optical density at a wavelength of 600 nm using an automated growth curve detector (Bioscreen C, Finland). Briefly, overnight cultures were diluted (1:200) and incubated at 37°C with shaking at 220 rpm. The OD_600_ of the bacterial culture was measured at 1 h intervals for 24 h.

### Antimicrobial susceptibility testing

Susceptibility of *S. aureus* strains to various antibiotics was detected by the broth microdilution method and the disk-diffusion method according to the guidelines of American Clinical and Laboratory Standards Institute (CLSI). To determine the minimal inhibitory concentrations (MICs) of the antibiotics for *S. aureus,* two-fold dilutions of antibiotics in 96 wells microplates containing Mueller-Hinton broth were made to concentrations from 256 to 0.125 mg/L. Overnight cultures of the bacteria were adjusted to the 0.5 McFarland standards and inoculated 1:200 into the MH broth (for oxacillin, MHB + 2% NaCl) and then incubated at 35°C for 24 h. The lowest concentration inhibiting visible growth of the bacteria was recorded as MIC.

### Visualization of biofilm formation by confocal laser scanning microscopy

Overnight cultures of *S. aureus* strains were diluted with TSB supplemented with 1% glucose at a ratio of 1:200, then inoculated in cell culture dishes (23 mm diameter) with glass bottoms (FluoroDish, WPI, Florida, USA) and incubated at 37°C for 24 h. After removal of non-adhered cells, the biofilms were washed with phosphate-buffered saline, stained with a Live/Dead BacLight Viability Kit (Molecular Probes, Eugene, Oregon, USA), and subsequently analyzed with a Leica confocal laser scanning microscope (TCS SP8; Leica, Heidelberg, Germany) detecting the fluorescence intensities of SYTO9 and propidium iodide (PI). A series of images were acquired at 1 μm intervals in the Z section to measure the biofilm thickness. IMARIS 9.0 software (Bitplane, Zurich, Switzerland) was used to generate three-dimensional view of the biofilms.

To evaluate the effects of the antibacterial agents on 24-h biofilms, three biofilm-forming MRSA strains (strain USA300, strain 234 and strain 15098) were selected and cultured as described above for 24 h. After removal of the suspension cultures, four-fold MIC concentrations of the agents in fresh media were added and incubated at 37°C for an additional 24 h. The biofilms were then stained with the Live/Dead BacLight Viability Kit (Viable cells in the biofilms exhibited green fluorescence, and dead cells exhibited red fluorescence). The effects of the agents on 24-h biofilms were analyzed by visualizing three-dimensional biofilm structures with CLSM and calculating the ratio of dead/live bacterial cells with ImageJ software (Wayne Rasband, NIH, Bethesda, MD, USA).

### Expression and purification of recombinant ArlR and ArlS’

To construct the recombinant *S. aureus* ArlR and ArlS’ (histidine kinase domain of ArlS) expression plasmids (pET-SA*arlR* and pET-SA*arlS*), the DNA fragments were amplified from the genomic DNA of USA300 TCH1516 strain by PCR with the primers REarlR-f/REarlR-r and RarlSHK-f/RarlSHK-r (**Supplementary Table 1**), digested with XbaI/XhoI and NcoI/XhoI respectively, and inserted into a pET-28a(+) plasmid [29] at the corresponding sites. After transformation into BL21 (DE3), the bacteria were cultured in LB medium at 37°C for 4 h and incubated for another 12 h at 25°C with 0.4 mM isopropyl-1-thio-b-D-galactopyranoside. The cells were harvested, disrupted using sonication in lysis buffer (50 mM Tris-Cl and 300 mM NaCl, pH 8.0), and then centrifuged at 15,000 g for 15 min at 4°C. The recombinant His-tagged ArlR protein (rArlR) and histidine kinase domain of ArlS (ArlS’) in the supernatants were purified using affinity chromatography with a Ni-nitrilotriacetic acid column (Qiagen GmbH, Hilden, Germany) according to the manufacturer’s protocol.

### Inhibition assay for ArlS kinase activity

The inhibitory activity of oritavancin on the ATPase activity of the ArlS’ protein was measured using the Kinase-Glo^TM^ Luminescent Kinase Assay (Promega, Madison, WI, USA). The reactions were carried out in solid black, flat-bottomed 96-well plates. Briefly, 3 μg purified ArlS’ protein was pre-incubated with a series of dilutions of oritavancin in reaction buffer [40 mM Tris (pH 7.5), 20 mM MgCl_2_ and 0.1 mg/ml BSA] at 4°C for 30 min, then 4 μM ATP was added and incubated for 30 min at room temperature. Afterwards, an equal volume of the Kinase-Glo^TM^ Reagent was added to each well, mixed and kept at room temperature for 10 min before the final recording of the luminescence (RLU) with a Victor X5 Multilabel Plate Reader (PerkinElmer, Boston, Massachusetts, USA). The rate of inhibiting ATPase activity by oritavancin was calculated by the following formula:

$$\frac{\begin{array}{c} \mathrm{RLU}({\mathrm{ArlS}}^{\prime }+\mathrm{oritavancin}+\mathrm{ATP}+\mathrm{Reagent})\\ -\mathrm{RLU}({\mathrm{ArlS}}^{\prime }+\mathrm{ATP}+\mathrm{Reagent}) \end{array}}{\mathrm{RLU}(\mathrm{ATP}+\mathrm{Reagent})-\mathrm{RLU}({\mathrm{ArlS}}^{\prime }+\mathrm{ATP}+\mathrm{Reagent})}$$

### Checkerboard dilution assay

To detect if oritavancin and oxacillin shows synergism against MRSA strains, a synergism assay was performed using the checkerboard method [32]. Briefly, in order to prepare a range of drug concentrations which allows detection of antagonism, indifference/additive, and synergism, a series of twofold dilutions of oritavancin and oxacillin in 96-well microplates starting from the concentration of double the MIC were made to obtain combination of varying concentrations of the two antibiotics. Bacteria were prepared according to broth microdilution assay. The microplates were incubated overnight at 35°C and MIC was read as the least dilution without any turbidity. The combinational effect of the two antibiotics was defined according to the fractional inhibitory concentration (FIC) index, whereby FIC = MIC of oritavancin in combination/MIC of oritavancin alone + MIC of oxacillin in combination/MIC of oxacillin alone. The combination is considered synergistic when the FIC index is ≤0.5. Indifference was indicated by a FIC index >0.5 to ≤4, while antagonism when the FIC index is >4.

### RNA extraction and quantitative real-time (qRT)-PCR

For RNA extraction, the *S. aureus* strains were cultured at 37 °C with shaking for 4 or 10 h. The cell pellets were collected and washed with ice-cold normal saline and then homogenized using 0.1-mm Zirconia-silica beads in a Mini-BeadBeater-16 (Biospec, Bartlesville, USA) at a speed of 3,600 rpm for 1 min following cooling on ice for 1 min. This homogenization and cooling cycle were repeated five times, then the samples were centrifuged at 13,000 rpm and the bacterial RNA in the supernatant was purified using a RNeasy Mini kit (Qiagen) and quantified using an ND-1000 spectrophotometer (Nanodrop Technologies, Wilmington, USA). RNA samples that had a 260/280 ratio between 2.0 and 2.2 were reverse transcribed using an iScript cDNA synthesis kit (Bio-Rad) following the manufacturer’s protocol. The mRNA levels were quantified by using qRT-PCR with SYBR green PCR reagents (Takara, Japan) and the primers listed in **Supplementary Table 3**, using the housekeeping gene *gyrB* as an endogenous control. The amplification efficiency of all primer pairs was determined according to the standard curve with four magnitudes of templates. The specificity of primer pairs was determined with melting curve. All the qRT-PCR experiments were carried out in triplicate and the relative gene expression data were analyzed using the 2^−△△CT^ method [33].

### Promotor-reporter assay

To detect *mgrA* (locus tag: USA300HOU_0709) and *spx* expression in *S. aureus* strains, a green fluorescent protein -reporter assay was performed by using the shuttle vector pCM29 [28]. A fragment containing the putative *mgrA* promoter or *spx* promoter region was amplified from the genomic DNA of USA300 TCH1516 strain using corresponding primers listed **Supplementary Table 1**. The PCR products were digested with NheI and BamHI, and subsequently ligated to upstream of the GFP gene in pCM29 to generate the plasmids pCM29-*mgrA* and pCM29-*spx* respectively. Each plasmid was transformed into *E. coli* DC10B, then into *S. aureus* USA300 TCH1516 and Δ*arlRS* strains by electroporation. To monitor the *mgrA* and *spx* expression, *S. aureus* strains containing pCM29-*mgrA* or pCM29-*spx* were cultivated in TSB at 37°C with shaking respectively, and bacterial cultures were collected at different time points. After centrifugation, the pellets were washed three times with normal saline, resuspended and adjusted to OD_600_ = 1.0. The bacterial suspension was transferred to a black 96-well microplate and the fluorescence intensity was measured using a Victor X5 multilabel plate reader (PerkinElmer, Inc., USA) with excitation at 480 nm and emission at 515 nm. Values from quadruplicate wells were averaged, and the experiment was repeated at least once.

### Electrophoretic mobility shift assay (EMSA)

To analyze binding of the recombinant rArlR and the promoter regions of *spx*, EMSA were performed using the DIG Gel Shift Kit (Roche, Basel, Switzerland) according to the manufacturer’s instructions. Briefly, rArlR was phosphorylated prior to gel shift reaction by incubating ArlR with 50 mM acetylphosphate for 1 h. Meanwhile, the DNA fragment upstream *spx* (P*spx*) were amplified with corresponding primers listed in **Supplementary Table 1** and linked with digoxin-labelled dd-UTP respectively. The resulting DNA fragment, DIG-P*spx* was used as a probe which was loaded with increasing amounts of rArlR (0, 0.5, 1, 1.5 and 2 μg). The digoxin-labelled DNA fragment upstream *arlR* (P*arlR*) was used as a control. All samples were incubated at 25°C for 30 min. After electrophoresis on 6% non-denaturing polyacrylamide gel, the DNA fragments were transferred to positively charged nylon membranes (GE Healthcare Life Sciences, Pittsburgh, PA, USA) by electro-blotting and detected by an enzyme immunoassay following the manufacturer’s instructions.

## Results

### Effect of *arlRS* mutation in MRSA USA300 and USA500 strains on their susceptibility to oxacillin

The methicillin-resistant *S. aureus* USA300 FPR3757 strain (GenBank Accession Number: NC_007793) showed high resistance to oxacillin (MIC = 64 mg/L), while its isogenic mutant with transposon insertion in either *arlS* gene or *arlR* gene showed 4-fold to 8-fold decrease in MIC to oxacillin (MIC = 8 mg/L or 16 mg/L, **Supplementary Table 2**).

**Figure 1.**
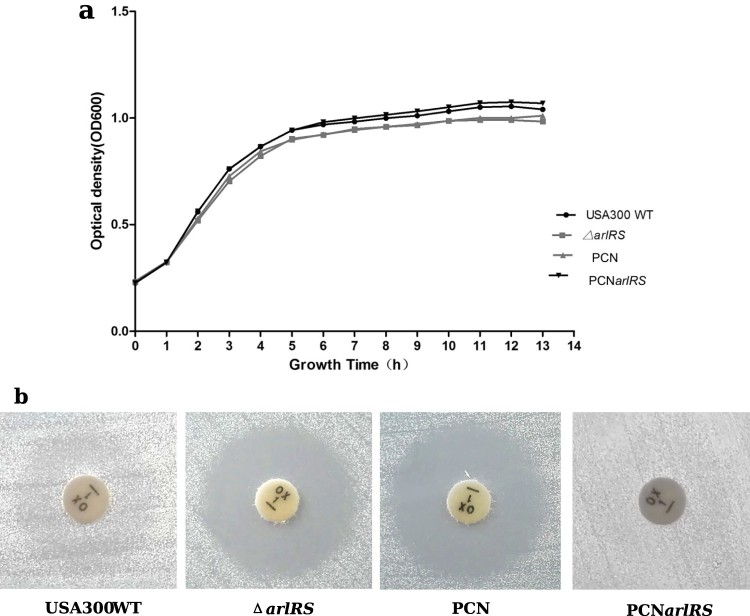
Effect of *arlRS* knockout on bacterial growth and oxacillin resistance of *S. aureus* USA300 strain. (a) Growth curves of *S. aureus* USA300 TCH1516 and its isogenic *arlRS* mutants. Bacterial strains were cultured in MHB + 2% NaCl at 35°C with shaking and the OD_600_ values were measured hourly using an automated growth curve detector. The experiments were repeated three times and a representative set of growth curves is shown. Δ*arlRS*- the *arlRS* knockout mutant; PCNarlRS −Δ*arlRS* complemented with plasmid expressing-*arlRS*; PCN −Δ*arlRS* transformed with an empty plasmid. (b) Oxacillin susceptibility of *S. aureus* USA300 TCH1516 and its isogenic *arlRS* mutants, detected by the Disk-diffusion method according to the CLSI guidelines.

To investigate the effect of ArlRS two-component system on oxacillin resistance in MRSA, an *arlRS* genes knockout mutant strain was constructed by homologous recombination using USA300 TCH1516 (GenBank Accession Number: NC_010079) as a parent strain, designated USA300Δ*arlRS.* The *arlRS* complementation strain PCN-*arlRS* was constructed by introducing a plasmid-expressing *arlRS* into the mutant. The wild type, mutant and complementation strains showed no obvious difference in bacterial growth in MHB + 2% NaCl at 35°C (Figure 1(a)). However, compared to the wild type strain, USA300Δ*arlRS* showed much higher susceptibility to oxacillin, as detected by disk-diffusion method (zone diameter = 20 mm vs 0 mm, shown in Figure 1(b)) and broth microdilution method (MIC = 64 mg/L vs 4 mg/L, shown in Table 2). Similarly, knocking out *arlRS* in MRSA USA500 strain 2395 (GenBank Accession Number: CP007499) resulted in much lower MIC to oxacillin (WT: 64 mg/L; USA500Δ*arlRS*: 8 mg/L). The altered phenotype was restored by *arlRS* gene complementation in both USA300Δ*arlRS* strain and USA500Δ*arlRS* strain (Table 2).

**Table 2.** The MICs of oxacillin for different S. aureus strains (the broth microdilution method).

	In USA300 Strain MIC (mg/L)	In USA500 Strain MIC (mg/L)
WT	64	64
Δ*arlRS*	4	8
Δ*arlRS*:: *arlRS*(Plasmid)	64	64
Δ*arlRS*:: PCN-spx	4	ND
Δ*arlRS*:: PCN-spx + CdCl_2_	64	ND
Δ*arlRS*:: PCN-trfA	4	ND
Δ*arlRS*:: PCN-trfA + CdCl_2_	16	ND
Δ*mgrA*	32	ND
WT:: pMX	64	64
WT:: pMX-spx	64	64
WT:: pMX + ATc	32	32
WT:: pMX-spx + ATc	<0.25	<0.125

### Effect of *arlRS* silence in clinical MRSA isolates on their susceptibility to oxacillin

In order to investigate the influence of ArlRS on oxacillin resistance in other clinical MRSA strains, the anti-sense RNA (asRNA) expression plasmids of *arlR* and *arlS* (pMX-*arlR,* pMX-*arlS*) were constructed and transformed to 6 clinical MRSA isolates, using the empty vector pMX6 as a negative control. The transformation was succeeded in three isolates with high oxacillin resistance (MICs ranging from 64 mg/L to 128 mg/L). With 250 ng/ml ATc induction, the three strains containing pMX-*arlR* or pMX-*arlS* showed in 2–4-fold decrease in MIC to oxacillin, while no obvious change was found in the vector control (**Supplementary Table 3**).

### Influence of *arlRS* knockout on expression of β-lactam resistance related genes

To define the *arlRS* regulon and the role of ArlRS in oxacillin resistance in MRSA, total RNAs from the wild-type USA300 TCH1516 strain and *arlRS* knockout mutant USA300Δ*arlRS* in mid-log growth phase (4 h) and stationary phase (10 h) were extracted, and transcriptional levels of β-lactam resistance related genes were determined by the RNA-Seq (Table 3). The mRNA levels of two transcriptional regulator genes, *spx* (locus tag: USA300HOU_0955) and *mgrA* (locus tag: USA300HOU_0709)*,* and a cell wall antibiotic resistance-related gene, *trfA* (locus tag: USA300HOU_0956) in USA300Δ*arlRS* were found to be down-regulated 4-fold, 3.7-fold and 1.3-fold respectively at 4 h, and 6.3-fold,5-fold and 1.3 fold respectively at 10 h, while no significant change was detected in transcriptional levels of other genes including *mecA* and *blaZ*.

**Table 3.** Comparison of the transcription level of genes related to β-lactam antibiotics resistance in USA300 and ΔarlR*S*.

ORF (USA300HOU_)	Annotation	Ratio (Mu/WT)	
		4h	10h
0031	MecA	1.01	0.86
0032	MecR1	0.97	0.79
pUSA300 HOUMR0011	BlaZ	0.99	0.93
pUSA300 HOUMR0012	BlaR1	0.93	1.09
pUSA300 HOUMR0013	BlaI	0.74	1.05
1309	FemA	0.86	1.25
1310	FemB	0.84	1.06
1121	penicillin-binding protein 1	1.04	1.02
1388	penicillin binding protein 2	1.09	1.14
1554	penicillin-binding protein 3	1.01	0.88
0662	penicillin-binding protein 4	1.06	1.84
1001	FmtA	1.24	1.02
2150	FmtB	6.36	3.01
2151	GlmM, phosphoglucosamine mutase	1.11	0.93
1294	FmtC	0.95	1.16
0663	AbcA	0.95	0.78
0956	TrfA, Cell Wall Antibiotic Resistance	0.78	0.79
0774	Llm, glycosyl transferase	0.91	1.25
2429	Flp protein, penicillin binding	0.87	1.02
2510	penicillinase repressor family protein	1.68	1.30
1693	metal-dependent hydrolase	1.17	0.83
0955	**Spx**	**0.25**	**0.16**
0709	**MgrA**	**0.27**	**0.20**
2059	SigB	0.95	1.31
2035	AgrA	0.98	0.83

**Figure 2.**
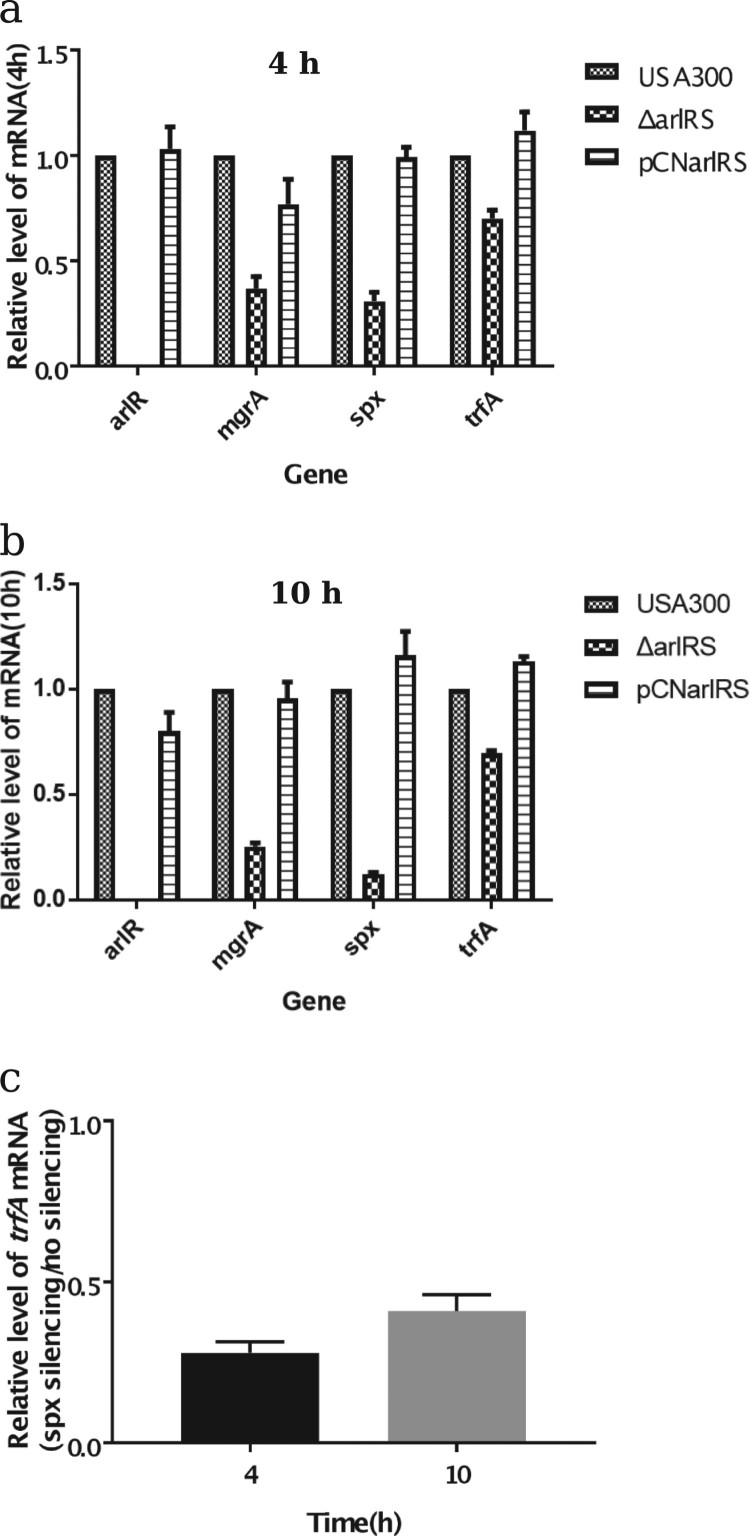
Effect of *arlRS* knockout on transcription of *spx*, *mgrA* and *trfA* (qRT-PCR). The mRNA in the *S. aureus* wild type strain (USA300), *arlRS* knockout mutant (Δ*arlRS*) and complementation strain (PCNarlRS) was extracted from 4 h (a) and 10 h (b) cultures. The transcription levels of *arlR, mgrA*, *spx* and *trfA* were quantified by qRT-PCR with SYBR green PCR reagents and the primers listed in Supplementary Table 3. The mRNA in the *S. aureus* USA300-pMX-*spx* was extracted from 4 and 10 h under no ATc induction and 250 ng/ml ATc induction conditions. The transcription levels of *trfA* was quantified by qRT-PCR (c). The relative expression levels of the genes are represented as mean ± standard deviation.

To confirm the RNA-Seq results, two different methods were carried out, including quantitative RT–PCR and the promoter-reporter system. The qRT-PCR result showed that in USA300Δ*arlRS spx*, *mgrA* and *trfA* transcription decreased 3.1-fold, 2.6-fold and 1.4-fold respectively at 4 h and decreased 6.1-fold, 4.3-fold and 1.4-fold at 10 h*,* while in the *arlRS* complementation strain those genes expression was restored (Figure 2(a,b)). Besides, by using promoter-GFP reporter assay, the intensity of GFP fluorescence indicating either *spx* or *mgrA* expression, was lower in USA300Δ*arlRS,* compared to that in the wild type strain (**Supplementary Figure 1**).

### Effect of *spx* and *mgrA* on MRSA susceptibility to oxacillin

To investigate the effect of *spx* and *mgrA* on oxacillin resistance in MRSA, a *spx* silence strain (USA300-pMX-*spx*) and an *mgrA* knockout mutant (USA300Δ*mgrA*) were constructed, using USA300 TCH1516 as a parent strain. In MHB + 2% NaCl medium, both USA300-pMX-*spx* and USA300-pMX6 (an empty vector control) strains had an oxacillin MIC of 64 mg/L; when 250 ng/ml ATc was added in the medium, the control strain USA300-pMX6 had an oxacillin MIC of 32 mg/L, while USA300-pMX-*spx* showed a significant decrease in oxacillin MIC (below 0.25 mg/L, Table 2). As a control, when 250 ng/ml ATc was present but oxacillin was absent in the medium, the OD_600_ value of USA300-pMX-*spx* culture was the same as that of USA300-pMX6 culture. Similar results were obtained in the *spx* silence strain of USA500. Meanwhile, the *mgrA* knockout mutant USA300Δ*mgrA* showed a 2 two-fold decrease in oxacillin MIC, compared to the wild type strain (32 mg/L vs. 64 mg/L).

To further investigate the role of *spx* in oxacillin susceptibility in the *arlRS* mutant, the cadmium chloride (CdCl_2_) inducible *spx* gene overexpression plasmid pCN*-spx* was constructed and introduced into USA300Δ*arlRS*. The MIC to oxacillin in USA300Δ*arlRS* containing pCN*-spx* was similar to that of the mutant when there was no CdCl_2_ in the culture medium, while it was restored to wild strain level by overexpressing *spx* when 2 μM CdCl_2_ was present for induction (Table 2).

### Regulation of *spx* expression by ArlRS

**Figure 3.**
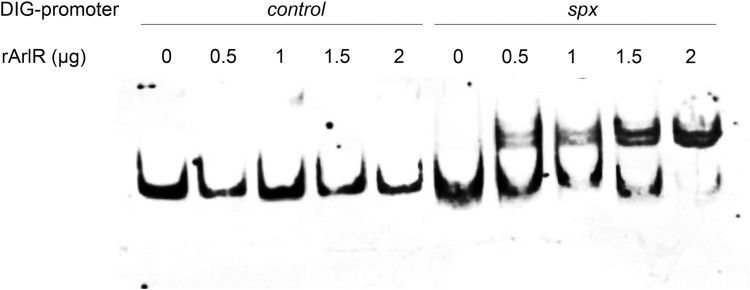
Binding of rArlR to the *spx* promoter region (EMSA). The promoter regions of *spx* and *arlR* (as a control) were amplified and labelled with digoxin (DIG). Lanes 1–5 were loaded with 0.4 ng DIG-*arlR* promoter region and increasing amounts of recombinant ArlR (0, 0.5, 1, 1.5, 2 μg respectively). Lanes 6–10 were loaded with 0.4 ng DIG-*spx* promoter region and increasing amounts of recombinant ArlR (0, 0.5, 1, 1.5, 2 μg respectively). The DIG-labelled DNA fragments were transferred to positively charged nylon membranes and visualized by an enzyme immunoassay using anti-Digoxigenin-AP, Fab-fragments and the chemiluminescent substrate CSPD. Chemiluminescent signals were recorded on X-ray film.

To investigate whether ArlRS regulates *spx* expression directly, EMSA were performed with a digoxin labelled 146-bp DNA fragment upstream of *spx* (P*spx*, 263-bp to 118-bp upstream of the start codon of *spx*) and recombinant the DNA binding protein ArlR (rArlR). As shown in Figure 3, when 0.5 μg to 2 μg rArlR was present, it bound to P*spx* and formed a DNA–protein complex, shifting P*spx* behind (lane 6 to lane 10) in a dose-dependent manner, compared to P*spx* alone (lane 1). However, as a control, up to 2 μg rArlR did not shift a 150-bp DNA fragment upstream of *arlR* (P*arlR*) behind (lane 2 to lane 5).

### Inhibition of ArlS’ kinase activity

In order to screen for compounds that can block the histidine kinase ArlS autophosphorylation and subsequent signal transduction, the recombinant cytoplasmic domain of ArlS protein (ArlS’) which contains the HATPase_c domain was purified and its activity to hydrolyze ATP was confirmed by the Kinase-Glo™ Luminescent Kinase Assay (**Supplementary Figure 2a**). By screening in the FDA-approved drugs library, oritavancin diphosphate, was found to inhibit the kinase activity of ArlS’ (3 μg) by 98% at a concentration of 25 μM. A dose-dependent inhibition was further detected and the IC_50_ value of oritavancin was 5.47 μΜ, calculated with the logistic regression fit (**Supplementary Figure 2b**).

**Figure 4.**
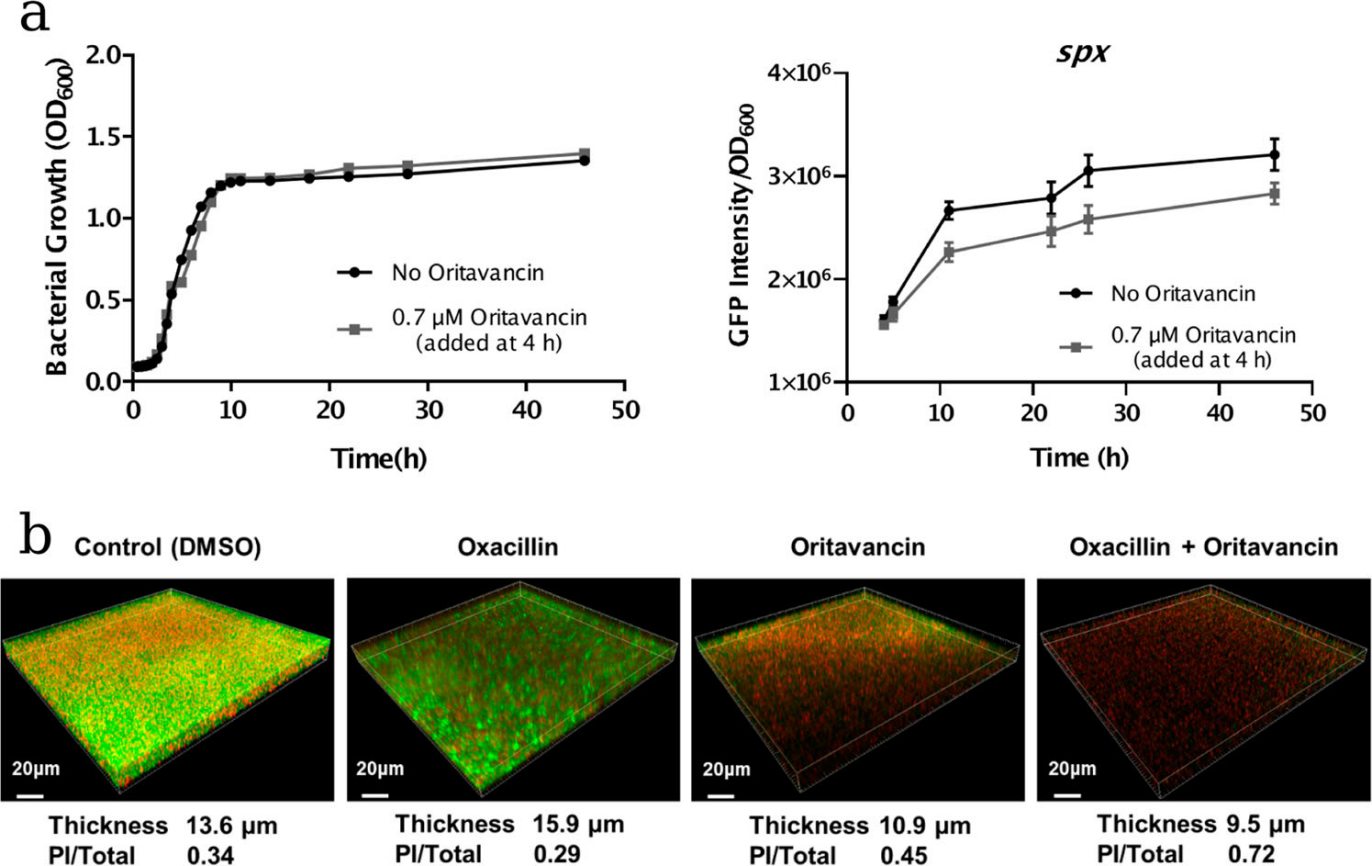
Effects of oritavancin on *spx* expression and oxacillin susceptibility of USA300 in mature biofilms. (a) The effect of oritavancin on *spx* expression was detected with *spx* promoter-GFP reporter assay: the USA300-P*spx* strain was cultivated to mid-log phase (4 h, OD_600_ ∼0.6), then oritavancin was added to a final concentration of 0.7 μM. The cell densities and GFP fluorescence intensities were monitored at different time points during cultivation. no antibiotic treated culture was used as a control. The *spx* expression is indicated with the intensity of GFP fluorescence normalized with OD_600_ value. (b) Biofilms of the MRSA USA300 strain TCH1516 were grown in fluorodishes at 37°C for 24 h. Afterwards, the planktonic cells were removed and fresh TSB containing 0.1% DMSO (control), 32 mg/L oxacillin, 3.1 μM oritavancin, or 32 mg/L oxacillin plus 3.1 μM oritavancin was added and incubated at 37°C for another 24 h. The mature biofilms were stained with SYTO9 and propidium iodide (PI) and observed under a Leica TCS SP8 CLSM with a 63× 1.4-NA oil-immersion objective. The viable cells were stained with green fluorescence while the dead cells with red fluorescence. Images of three-dimensional biofilm structure were constructed using IMARIS 9.0 software and the thicknesses of the biofilms were shown. The fluorescence intensities of SYTO9 and PI were determined by using Image J software, and ratio of PI/Total (PI + SYTO9) were calculated.

To investigate the effect of oritavancin on *spx* expression, the *spx* promoter-GFP reporter assay was carried out. The USA300-P*spx* strain was cultivated to mid-log phase (4 h), then oritavancin was added to the culture to a concentration of 0.7 μM and no antibiotic treated culture was used as a control. Afterwards, the cell densities and GFP fluorescence intensities were monitored at different time points during cultivation. It showed that during 42 h cultivation, *spx* expression (indicated with the intensity of GFP fluorescence normalized with OD_600_ value) in oritavancin treatment group was lower than that in the control group (Figure 4(a)).

### Effect of oritavancin on the oxacillin susceptibilities of the MRSA strains in planktonic state and mature biofilms

To detect if oritavancin enhances oxacillin susceptibilities in MRSA strains in planktonic state, a synergism assay was performed using the checkerboard method. Firstly, the MICs of oritavancin and oxacillin were detected and confirmed in USA300, USA500 strains and three clinical MRSA isolates; then the combinational effect of the two drugs was determined and the fractional inhibitory concentration (FIC) index was calculated. The FIC index varied from 0.375 to 0.5 in five MRSA strains, all defined as synergism effects (Table 4).

**Table 4.** Effects of combination of oritavancin and oxacillin against MRSA strains.

MRSA strains	MIC			FIC index	Effect
	Oxacillin (mg/L)	Oritavancin (μM)	Oxacillin (mg/L) +Oritavancin (μM)		
USA300	64	0.78	16 + 0.195	0.5	synergism
USA500	64	0.78	16 + 0.195	0.5	synergism
234	128	1.56	32 + 0.195	0.375	synergism
15098	128	0.78	32 + 0.195	0.5	synergism
184749	64	0.78	16 + 0.195	0.5	synergism

As *S. aureus* biofilm formation is associated with antibiotic resistance, we further investigated the effect of oritavancin on oxacillin susceptibilities of bacterial cells in biofilms. The MRSA strain USA300 was inoculated in the fluorodishes and incubated for 24 h to form mature biofilms. Afterwards, 0.1% DMSO (control), 32 mg/L oxacillin, 3.1 μM oritavancin, 32 mg/L oxacillin plus 3.1 μM oritavancin was added respectively and incubated for another 24 h. After Live/Dead staining with SYTO9 and propidium iodide (PI), the thicknesses of mature biofilms and viability of embedded cells of MRSA were detected by Confocal Laser Scanning Microscopy. It showed that in control group, oxacillin group, oritavancin group and drug combination group, the average thickness of biofilm was 13.6, 15.9, 10.9 and 9.5 μm, respectively, while the ratio of dead cells in the treated mature biofilms indicated with PI/(PI + SYTO9) fluorescence intensity was 0.34, 0.29, 0.45 and 0.72, respectively (Figure 4(b)).

## Discussion

In this work, we find that the two-component signal transduction system ArlRS regulates susceptibility to oxacillin in MRSA strains USA300, USA500 as well as three clinical isolates. Furthermore, oritavancin diphosphate was found to inhibit the kinase activity of ArlS’ and have synergy effect on antibacterial activities of oxacillin against the MRSA strains.

Transposon insertion mutation in USA300 *arlR* or *arlS* gene, *arlRS* knockout in USA300 and USA500, silencing *arlR* or *arlS* in MRSA isolates, all result in increased sensitive to oxacillin, suggesting an important role of *arlRS* in regulating oxacillin resistance in MRSA. Transcriptional profiles analysis of USA300 and its *arlRS* knockout mutant Δ*arlRS* shows no significant difference in mRNA levels of the genes that have been reported to be related to β-lactam antibiotics resistance, except *mgrA* and *fmtB*. MgrA is a global transcriptional regulator, which has been found to negatively regulate autolysis genes and affect expression of virulence genes in *S. aureus* [34–36]. MgrA acts as a direct activator for *abcA*, a gene encoding an ATP-dependent transporter, which is related with cell wall autolysis and β-lactam antibiotics resistance [37]. Besides, *mgrA* deletion in *S. aureus* ISP794 strain resulted in a decrease in oxacillin MIC from 4 to 2 mg/L [38]. ArlRS has been reported to modulate *mgrA* expression. However, the results from previous reports and in the present study indicate *mgrA* is not the major factor affecting oxacillin susceptibility in the *arlRS* mutant. First, although *mgrA* may affect cell wall autolysis, Memmi G et al. have demonstrated that inactivation of *arlRS* does not play a role in autolysis of MRSA strains including USA300 [16]. Meanwhile, in the present study, *abcA* expression shows no significant change in *arlRS* mutant, compared to USA300 wild-type strain. Furthermore, we find that *mgrA* knockout in USA300 only results in a slight reduction of MIC for oxacillin (from 64 mg/L to 32 mg/L), while knockout *arlRS* in USA300 leads to a much lower MIC value (4 mg/L). There is limited information about another oxacillin resistance gene *fmtB*, which codes for a ∼263 kDa cell wall-anchored protein. Komatsuzawa H et al. have found that transposon insertion mutation in *fmtB* of MRSA strain COL is indirectly linked with loss of oxacillin resistance, which cannot be restored by trans-complementation with *fmtB* in *fmtB* mutants but can be restored by overexpressing its downstream *glmM* gene [39]. In this work, however, the *glmM* expression showed no difference in the USA300 and Δ*arlRS* strains. The increased *fmtB* mRNA levels in Δ*arlRS* can be explained by the decreased *mgrA* expression, since MgrA represses *fmtB* transcription [34].

For the first time, we demonstrate that *spx*, which has not been reported to be associated with oxacillin susceptibility before, plays a great role in ArlRS mediated oxacillin resistance. First, the mRNA level of *spx* gene is dramatically decreased in the Δ*arlRS,* compared to that in USA300. Besides, overexpressing *spx* in Δ*arlRS* restored the oxacillin resistance to a similar level in USA300 (MIC = 64 mg/L). Furthermore, the response regulator ArlR can direct regulate *spx* transcription by binding to its promoter region (Figure 3 and **Supplementary Figure 3**). More importantly, silencing *spx* results in a dramatic increase of oxacillin susceptibility in both USA300 and USA500 strains (MIC <0.25 and 0.125 mg/L respectively). Spx is first reported in *Bacillus subtilis* as a global regulator of genes that is induced by disulfide stress, through a unique mechanism that requires direct interaction with the subunit of RNA polymerase but not with DNA [40]. Spx is highly conserved in low G + C Gram-positive bacteria such as *Staphylococcus, Listeria, Enterococcus,* and *Streptococcus* [41–43]. However, the function of *spx* in *Staphylococcus* has not been intensively studied. Recent works tend to recognize *spx* as an essential gene in both *S. aureus* and *S. epidermidis* [41,42], although it can be knocked out in *B. subtilis.* Pamp SJ et al. have reported construction of a *spx* deletion mutant in the *S. aureus* 8325-4 strain, and find inactivation of *spx* makes the cells highly susceptible to multiple stresses including high and low temperature, salt stress, and hydrogen peroxide [42]. Villanueva M et al. have discovered recently by deep sequencing that the Δ*spx* strain harbours suppressor mutations that allowed it to grow without *spx* [44]. Our group has tried to construct a *spx* deletion mutant of USA300 but cannot achieve. Thus, we use antisense RNA technique to conditionally silence *spx* expression in USA300 and USA500 with ATc. Compared to no ATc induction condition, 250 ng/ml ATc induced *spx* antisense RNA expression results in delayed bacterial growth in lag phase and log phase, and similar levels in stationary phase. Meanwhile, *spx* silencing leads to an over 256-fold increase in susceptibility to the oxacillin and *spx* overexpression can restore loss of oxacillin resistance in Δ*arlRS*, suggesting its important role in MRSA. There are very limited reports about the relation of Spx and antibiotic resistances. Jousselin A et al. have found Spx modulates the expression of *trfA* gene involved in glycopeptide resistance in *S. aureus* [45]. The *trfA* encodes teicoplanin resistance factor A, which closely resembles an adaptor protein of *Listeria monocytogenes* and *B. subtilis*. Deletion of *trfA* and *trfB* (Δ*trfAB*) in MRSA strain NRS3 leads to increased susceptibility to oxacillin [46]. Our qRT-PCR data is consistent with the finding, for the transcription level of *trfA* is decreased in the Δ*arlRS* by ∼1.4 fold and is restored in *arlRS* complementation strain. In the *spx* silencing strain, the mRNA levels of *trfA* at 4 and 10 h were decreased by 3.6-fold and 2.5-fold (Figure 2(c)). Furthermore, the CdCl_2_ inducible *trfA* gene overexpression plasmid pCN*-trfA* was constructed and transformed to USA300Δ*arlRS*. The MIC to oxacillin in USA300Δ*arlRS* containing pCN*-trfA* was similar to that in the mutant when there was no CdCl_2_, while it was partially restored by overexpressing *trfA* when 2 μM CdCl_2_ was present (Table 2). These results indicate that *trfA* plays a role in *arlRS* and *spx* mediated oxacillin susceptibility. Besides, Göhring N et al. show that inactivation of *yjbH*, which encodes a Spx-interacting protein governing Spx proteolytic degradation, led to moderate resistance to oxacillin in *S. aureus* [47]. However, no change in *yjbH* mRNA level was found in Δ*arlRS* in our work. Thus, the role of *spx* in regulating oxacillin resistance warrants further investigations, e.g. whether *spx* mediates oxidative stress responses to hydroxyl radicals produced by bactericidal antibiotics.

The important role of ArlRS in regulating oxacillin resistance in MRSA strains indicates that this two-component system is a potential target for antimicrobial resistance breaker drugs, which may enhance the antibacterial activity of oxacillin. Bacterial two-component systems (TCSs) have been demonstrated to be promising targets [48–50], not only because they are vital to bacterial survival, virulence and antibiotic resistance, but also because their homologues have not been identified in humans. Novel inhibitors have been developed against several bacterial TCSs, including *Enterococcus faecium* VanSR (vancomycin resistance) [51], *Staphylococcus epidermidis* WalKR (bacterial growth and biofilm formation) [52], *E. coli* QseCB (virulence) [53] and *Mycobacterium tuberculosis* DosRST (persistence) [54]. Most of the inhibitors are screened or designed against histidine kinase (HK) CA (HATPase_c) domain because of its conserved features and its essential role in TCS signal transduction [55]. In this work, by screening in the FDA approved drugs library, we have found that oritavancin (diphosphate) can inhibit the ATPase activity of the recombinant histidine kinase ArlS CA domain. Oritavancin is a semisynthetic lipoglycopeptide analogue of vancomycin, which has multiple mechanisms of action against exponentially growing *S. aureus* cells, including the inhibition of cell wall synthesis and RNA synthesis, disruption of membrane potential and increasing membrane permeability [56,57]. Our finding suggests that inhibition of ArlS kinase activity by oritavancin thereby interfering the signal transduction may be another mechanism of action against MRSA, because ArlRS modulates oxacillin resistance. This may be one of the reasons for the fact that oritavancin has synergy effect on antibacterial activities of oxacillin against the MRSA strains (Table 4). Furthermore, CLSM observation showed that ArlRS regulated MRSA biofilm formation (**Supplementary Figure 4**): compared to the parent strain USA300, USA300Δ*arlRS* formed thinner biofilm layers (10.32 μm VS. 17.83 μm), which was restored by *arlRS* complementation (PCN*arlRS*, 17.8 μm) but not by the empty vector (PCN, 11.26 μm). This may give a possible explanation for that oritavancin treatment results in disruption of mature MRSA biofilms and facilitates bactericidal activity of oritavancin and oxacillin against embedded *S. aureus* cells (Figure 4). Our findings are consistent with the report by Belly A et al. that oritavancin is active against biofilm *S. aureus* cells *in vitro* [58].

In conclusion, our work demonstrates the two-component system ArlRS plays vital roles in regulating oxacillin susceptibility via direct modulation of *spx* expression and in regulating biofilm formation in MRSA strains. ArlRS is an attractive target for breaking oxacillin resistance of MRSA in planktonic state and in mature biofilms.

## Supplementary Material

Supplemental Material
