# Supplementary material for: The role of ArlRS in regulating oxacillin susceptibility in methicillin-resistant Staphylococcus aureus indicates it is a potential target for antimicrobial resistance breakers
Source: Emerg Microbes Infect. 2019 Mar 29;8(1):503–15. doi: 10.1080/22221751.2019.1595984 (PMC6455253; doi:10.1080/22221751.2019.1595984)
Supplement: Supplemental Material [file TEMI_A_1595984_SM1321.pdf]

Supplementary Information

Supplementary Table 1. Primers used in this study

Primers	Sequence 5'-3'	Restriction Site
**For construction of *arlRS* deletion mutant**		
arlRS-UF	GGGGTACCAAAAAACAAAAGCAGTAAACCTAAAG	KpnI
arsRS-UR	GACGTCTCAGTCATGACCTCATATTACGACTTTTTCTAA	
arlRS-DF	TTAGAAAAAGTCGTAATATGAGGTCATGACTGAGACGTC	
arlRS-DR	CGACGCGTGCATTGTACCGTATGAATCAG	MluI
**For construction of asRNA plasmids**		
asr*arlR*-F	GAAGATCTTCGTAATATGAGGTGTACAAATG	BglII
asr*arlR*-R	AGCGGCCGCCGTCTTGTCCATCATACTC	EagI
asr*arlS*-F	GAAGATCTCAAAACGTAAATTGCGCAAT	BglII
asr*arlS*-R	AGCGGCCGCTTCGTTCTGCATCATCAA	EagI
as*spx*-F	GAAGATCTCAAATAGGAGAGTGAGATGTATG	BglII
as*spx*-R	AGCGGCCGCATCAATTGTTAAATGTTCAGAA	EagI
**For construction of GFP reporter plasmids**		
SA-*spx*-pro-GFP-F	CTCGCTAGCCACTAGGTTGGATGCCTG	NheI
SA-*spx*-pro-GFP-R	CTCGGTACCTCATTGCTTAAAATTTAGTTATAGATCAAG	BamHI
SA-*mgrA*-pro-GFP-F	CACGCTAGCCGTTAATTATGGATATTCTTATTTTTCATC	NheI
SA-*mgrA*-pro-GFP-R	CACGGTACCCAGACATACTATCCGTTTTTTTCTC	BamHI
**For construction of complemented plasmids**		

SA-*arlRSc*-R	GTTGGATCCTGACTTTGATTGACGTCTCAGT	BamHI
SA-*arlRSc*-F	GTTGTCGACTGTGTGAAATGTTTAAACTACGGT	SalI
SA-*spx*-RBS-F	CTCGGTACCTAGGAGAGTGAGATGTATGGTAAC	BamHI
SA-*spx*-RBS-R	CTCGAATTCTTAGTCAACCATACGTTGTGC	EcoRI
SA-*trfA*-RBS-F	CTCGGTACCTGTAAGGAGTGAGATGATATGAGA	BamHI
SA-*trfA*-RBS-R	CTCGGTACCATCGTTATTCAGTTGTCTCTGG	KpnI
**For detection of gene expression by qPCR**		
SA-*arlR*-qRT-S	TTCTTCAATATCAAACGGCTTA	
SA-*arlR*-qRT-R	GACAACAATCTACACCTAT	
SA-*mgrA*-qRT-S	AACGAATGGAACAAGTAG	
SA-*mgrA*-qRT-R	ACCTAATAAGCGATTAAGTT	
SA-*spx*-qRT-S	GTTGATATTGATTCACTACCA	
SA-*spx*-qRT-R	CGTTGTGCTTCTTGTAAT	
SA-*trfA*-qRT-S	ATCGAGGCCCGTGGATTTAG	
SA-*trfA*-qRT-R	TCGACACCTTTTTCAAAGGCA	
**For protein expression**		
REarlR-F	TCGTCTAGAAAAAGTCGTAATATGAGGTGTAC	XbaI
REarlR-R	TCGCTCGAGTCGTATCACATACCCAACGC	XhoI
RarlSHK-f	TCGCCATGGTTGAAGATGCGTCACATGA	NcoI
RarlSHK-r	TCGCTCGAGAAATATGATTTTAAACGTTGTTCCTTTG	XhoI
**For nucleotides amplification for EMSA**		
SA-*spx*-pro-short-F	TAATTATCATTATAACAGTATTTCTTAAAAATGTAAG	
SA-*spx*-pro-short-R2	CATTAAAATTAAATGAGAAAAACCTA	
SA-*arl*-pro-short-F	GAGTTAATAAATAATTAATGATTGTAGCT	
SA-*arl*-pro-short-R	ACTTTTTCTAATAAGGTAATATATTTTAAATTTTG	

Supplementary Table 2. The MICs (mg/L) of antibiotics for USA300 and its transposon insertion mutants (the broth microdilution method)

Antibiotics	USA300 WT	USA300 *-arlS*	USA300 *-arlR*	Quality Control ATCC29213
Oxacillin	64	8	16	0.25
Vancomycin	2	4	2	2
Teicoplanin	1	1	1	1
Linezolid	4	2	2	2
Daptomycin	1	2	1	1
Rifampicin	0.03	0.25	0.06	0.015
Gentamicin	0.5	8	2	1
Minocycline	0.25	2	0.25	0.25

Supplementary Table 3. The MICs for Oxacillin in the clinical MRSA strains

	**MRSA Strain 1 234**	**MRSA Strain 2 15098**	**MRSA Strain 3 184749**
pMX	128	128	64
pMX-arlR	128	128	64
pMX-arlS	128	128	64
pMX + ATc	64	128	64
pMX-arlR + ATc	32	32	16
pMX-arlS + ATc	32	64	16

Supplementary Figure 1

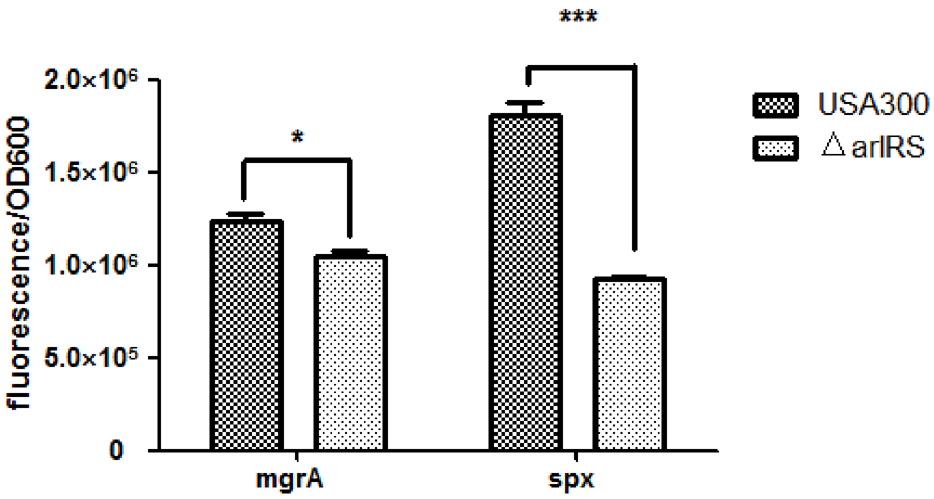

Supplementary Figure 1. Effect of *arlRS* knockout on transcription of *spx* and *mgrA* (promoter-GFP reporter assay)

A ~200 bp fragment containing the putative *mgrA* promoter or *spx* promoter region was amplified from the genomic DNA of USA300 TCH1516 strain and inserted into upstream of the GFP gene in pCM29. The resulting plasmids pCM29-*mgrA* and pCM29-*spx* were transformed into USA300 and Δ*arlRS* strains by electroporation. The intensity of GFP fluorescence indicating either *spx* or *mgrA* expression was monitored with excitation at 480 nm and emission at 515 nm. The experiment was performed in quadruplicates and was repeated at least once. The representative result at 4 h was shown as mean values ± standard derivations.

Supplementary Figure 2

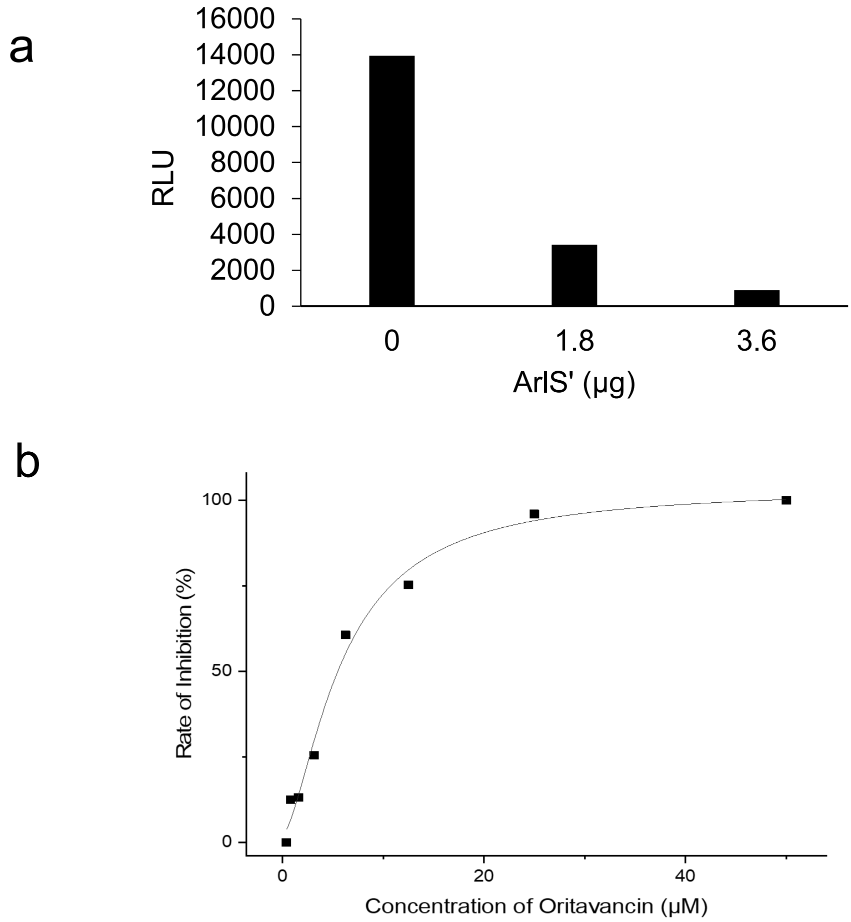

Supplementary Figure 2. Inhibition of ArlS kinase activity by oritavancin

(a) Detection of ATPase activity of the purified recombinant cytoplasmic domain of ArlS protein (ArlS') using the Kinase-Glo™ Luminescent Kinase Assay. Briefly, 1.8 μg and 3.6 μg purified ArlS' was incubated with 4 μM ATP in 50 μl reaction buffer [40 mM Tris (pH 7.5), 20 mM $MgCl_2$ and 0.1 mg/ml BSA] for 30 min at room temperature. Afterwards, 50 μl Kinase-Glo™ Reagent was added, mixed and kept at room temperature for 10 min before the final recording of the luminescence (RLU). The reactions were carried out in solid black, flat-bottomed 96-well plates.

(b) Measurement of the inhibitory effect of oritavancin on the ArlS' ATPase activity. In brief, 3 μg purified ArlS' protein was pre-incubated with a series of dilutions of oritavancin (0.195, 0.39, 0.78, 1.56, 3.13, 6.25, 12.5, 25 and 50 μM) in reaction buffer at 4°C for 30 min, then 4 μM ATP was added and incubated for 30 min at room temperature. The dose-dependent inhibition of oritavancin on the ArlS' ATPase activity was measured with the Kinase-Glo™ Luminescent Kinase Assay, and the IC_{50} value of oritavancin was calculated with the logistic regression fit using the Origin 7.0 software.

Supplementary Figure 3

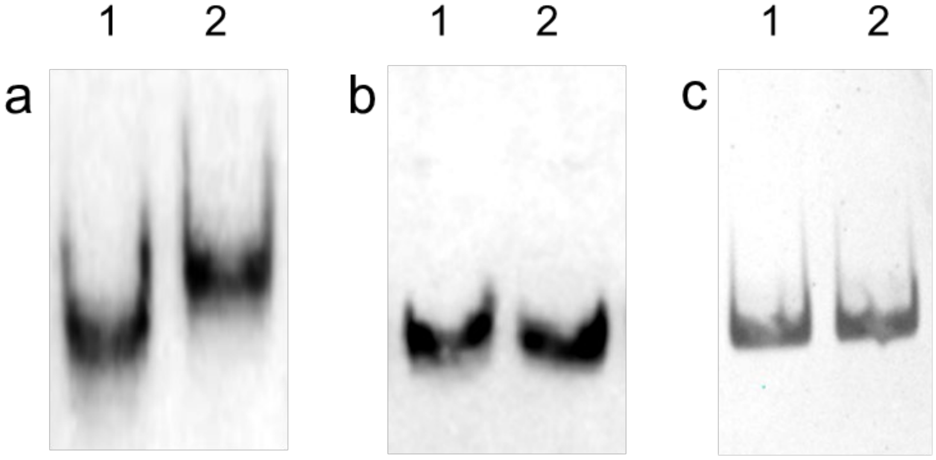

Supplementary Figure 3. Binding of rArlR to the different regions upstream of *spx* gene (EMSA)

(a) The 202 bp DNA fragment (263 bp to 62 bp upstream of the start codon of *spx*) was amplified, labeled with digoxin (DIG) and mixed with 0 and 2 μg recombinant ArlR respectively (lane 1-2). **(b)** The 98 bp DNA fragment (329 bp to 232 bp upstream of the start codon of *spx*) was amplified, labeled with digoxin (DIG) and mixed with 0 and 2 μg recombinant ArlR respectively (lane 1-2). The DIG-labeled DNA fragments were transferred to positively charged nylon membranes and visualized by an enzyme immunoassay using anti-Digoxigenin-AP, Fab-fragments and the chemiluminescent substrate CSPD. Chemiluminescent signals were recorded on X-ray film. **(c)** The 98 bp DNA fragment (129 bp to 33 bp upstream of the start codon of *spx*) was amplified by PCR. The 20 ng DNA fragment was mixed with 0 and 2 μg recombinant ArlR respectively (lane 1-2). After electrophoresis in a native PAGE, the gel was stained with GelRed.

Supplementary Figure 4

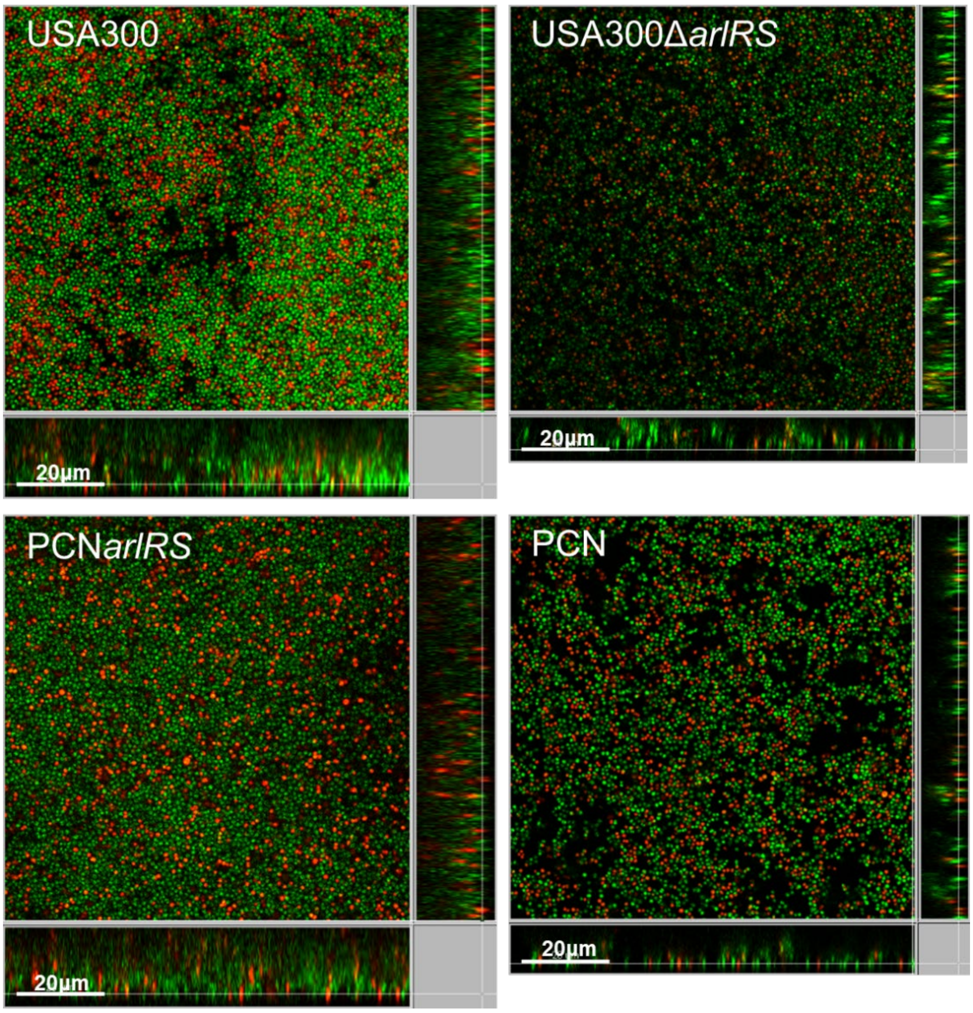

Supplementary Figure 4. The effect of *arlRS* genes knockout on biofilm formation of USA300 strain by confocal laser scanning microscopy

Overnight cultures of *S. aureus* strains (**USA300** -the USA300 TCH1516 wild type strain; **USA300Δ*arlRS*** **-**the *arlRS* knockout mutant*;* **PCN*arlRS*** -the *arlRS* complementation strain; **PCN -**a empty vector complementation strain for control) were diluted with TSB supplemented with 1% glucose at a ratio of 1:200, then inoculated in cell culture dishes with glass bottoms and incubated at 37°C for 24 h. After removal of planktonic cells, the biofilms were washed with PBS, stained with a Live/Dead BacLight Viability Kit, and subsequently analyzed with a Leica TCS SP8 confocal laser scanning microscope. A series of images were acquired at 1 μm intervals in the Z section to measure the biofilm thickness.
